# The role of astrocytes in ischemic stroke - mechanisms, functions and treatment

**DOI:** 10.3389/fcell.2025.1700564

**Published:** 2025-12-10

**Authors:** Yunlong Wang, Ding Wang, Dongyu Li, Yuewen Ma

**Affiliations:** 1 Department of Rehabilitation, The First Affiliated Hospital of China Medical University, Shenyang, China; 2 Department of Vascular Surgery, The First Affiliated Hospital of China Medical University, Shenyang, Liaoning, China

**Keywords:** ischemic stroke, astrocytes, inflammation, blood-brain barrier, combination therapy

## Abstract

Ischemic stroke, characterized by localized cerebral hypoperfusion due to various etiologies, exhibits high global incidence and mortality rates. Astrocytes, as key structural and functional components of the neurovascular unit, play a critical role in maintaining homeostasis in the central nervous system. Following ischemic stroke, astrocytes undergo activation and adopt a distinct phenotype known as reactive astrocytes, which exert complex and dual roles in the pathological process. Upon ischemic injury, astrocyte activation often exacerbates damage and impairs functional recovery; however, it can also confer neuroprotection by shielding neurons from injury and releasing trophic factors to facilitate repair. Importantly, due to the intricate cascade of events and mechanisms involved after ischemic stroke, the precise role of astrocytes remains challenging to definitively elucidate. Nonetheless, astrocytes represent a pivotal target for therapeutic interventions in ischemic stroke. Herein, we review the activation of astrocytes following ischemic stroke, focusing on the intrinsic molecular mechanisms and functional implications of astrocyte reactivity. Additionally, we discuss the potential of combining rehabilitation technologies to enhance drug delivery and therapeutic efficacy, offering a promising strategy for the treatment of ischemic stroke.

## Introduction

### Ischemic stroke

The onset of disease often imposes profound impacts on families and societies, with cerebrovascular diseases being particularly prominent due to their high mortality and disability rates. These diseases are primarily classified into transient ischemic attack, ischemic stroke (IS), intracerebral hemorrhage, and subarachnoid hemorrhage (Author Anonymous, 2019). Among these, IS not only the second leading cause of death worldwide after ischemic heart disease (GBD, 2019 Stroke Collaborators, 2021), but also has led to a continuous increase in the burden of stroke in China amid population aging. Since 2015, IS has become the leading cause of death and disability in China (Jing et al., 2023; Tu et al., 2023a). IS accounts for more than 86.9% of all stroke cases and is one of the major factors contributing to disability among Chinese residents. Current treatment strategies include thrombolytic therapy, mechanical thrombectomy, and bridging therapy (Tu et al., 2023b). Despite these interventions, more than 80% of IS patients still experience varying degrees of functional impairment that affects their daily activities (Crichton et al., 2016). Numerous studies have revealed a close relationship between astrocytes and the occurrence and progression of IS. For example, histological sections clearly show significant morphological changes in astrocytes after IS onset, which were later identified as signs of an activated state. These morphological alterations are inseparable from functional changes involving immune response, cerebrovascular structural remodeling, and cellular signaling (Sarraj et al., 2024; Tao et al., 2020; Le et al., 2021; Dhanesha et al., 2022). These findings indicate that the role of astrocytes in IS far more complex than previously understood. In-depth research on the relationship between astrocytes and IS will help explore novel approaches for improving outcomes of the disease.

### Astrocytes

The cellular composition of the brain is primarily divided into two major categories: neurons and neuroglial cells. Astrocytes, a type of neuroglial cell, are the most numerous and widely distributed glial cells in the brain (Jessen, 2004). Since the development of a novel staining method—silver chromate staining—by Golgi and others in 1886, the complexity and diversity of astrocytes have been progressively revealed (Somjen, 1988). Astrocytes exhibit a sophisticated morphology, typically appearing fusiform with 5–10 primary branches originating from the cell body. These branches extend and gradually subdivide into finer processes, leaflets, and end-feet (Bushong et al., 2002; Diniz et al., 2016). On average, each astrocyte envelops at least four neurons and forms connections with over fifty thousand excitatory synapses to facilitate signaling (Halassa et al., 2007; Chai et al., 2017). In addition to their close association with neurons, astrocytes extend end-feet that intimately contact at least one blood vessel (Hösli et al., 2022). Numerous astrocytes ensheath cerebral microvessels with almost no gaps, and their overlapping end-feet form a protective and supportive sleeve around the vasculature (Mathiisen et al., 2010; Wang et al., 2021). Owing to their intricate morphological architecture and intimate contact with both neural and vascular elements, astrocytes play crucial roles in the brain. Broadly speaking, they are involved in regulating cerebral blood flow, forming and supporting the blood-brain barrier (BBB), providing nutritional and metabolic support to neurons, and facilitating energy supply (Canfield et al., 2019; Mills et al., 2022; Gonzalez-Fernandez et al., 2020; Chuquet et al., 2010; Magistretti and Allaman, 2018).

### Changes in astrocytes during ischemic stroke

Under IS pathological conditions, astrocytes are activated by various stimuli and subsequently exhibit diverse phenotypic states—a condition referred to by Escartin et al. as “reactive astrocytes” (Escartin et al., 2021). Although activated astrocytes demonstrate complex phenotypes and functions, their roles may shift depending on the disease phase and interventions applied.

Reactive astrocytes primarily contribute through proliferation and cytokine secretion. Focal proliferation of astrocytes, along with microglia and pericytes, facilitates the formation of a protective barrier at the injury boundary, isolating damaged, inflamed, and fibrotic tissues from healthy tissue. This containment limits the spread of inflammation and injury, and progressively replaces impaired areas. These proliferative regions typically recede during recovery (Huang et al., 2014; Zhu et al., 2021; Zbesko et al., 2018). However, extensive astrocyte proliferation can lead to the formation of a permanent glial scar (Chen et al., 2022), which may impede axonal regeneration and hinder the reconstruction of neural networks, thereby limiting functional recovery. Additionally, reactive astrocytes secrete various cytokines. For instance, oxidative stress can stimulate astrocytes to release interleukin-10 (IL-10), which suppresses inflammatory responses and exerts neuroprotective effects (Segev-Amzaleg et al., 2013). IL-24, also secreted by astrocytes, participates in immune regulation by inhibiting the production of interleukin-6 (IL-6). Meanwhile, astrocytes upregulate neuroprotective mediators such as glutamate transporter-1 and cyclooxygenase-2 (COX-2) to mitigate neural damage (Feldman et al., 2015). On the other hand, astrocytic end-feet release angiogenesis-related factors such as vascular endothelial growth factor (VEGF) and transforming growth factor-β (TGF-β), which promote endothelial cell proliferation and contribute to vascular remodeling and stabilization (Zong et al., 2020). However, astrocyte activation also has detrimental effects: increased levels of interleukin-1β (IL-1β) may be linked to post-stroke cognitive impairment (Kim et al., 2020). IL-1β also stimulates nitric oxide (NO) secretion, and high concentrations of NO exhibit cytotoxicity and disrupt intercellular signaling (Yoo et al., 2008). Moreover, chemokines such as CCL2 and CXCL10 are among the earliest elevated factors in astrocyte-related immune responses and persist for several days, promoting inflammation and exacerbating brain injury (Grabarczyk et al., 2023).

**FIGURE 1:**
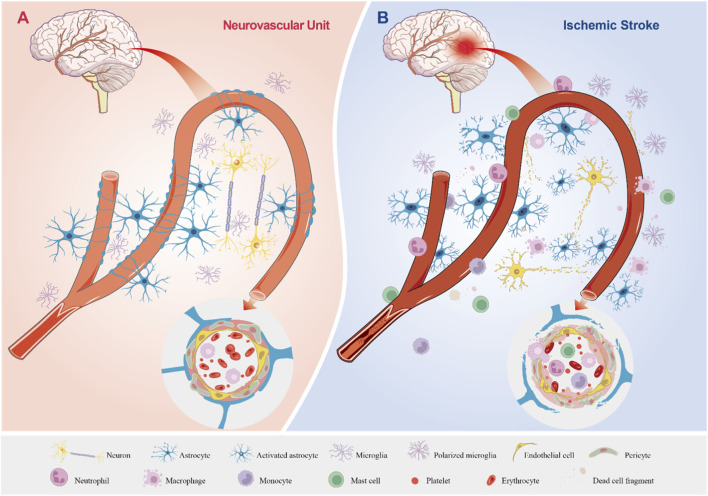
The Physiological State of the Neurovascular Unit and Its Pathological Changes in Ischemic Stroke. **(A)** The Neurovascular Unit Under Physiological Conditions. Astrocytes, neurons, and microglia display normal morphology and function. Astrocytes ensheath blood vessels, forming an integral component of the BBB, while establishing structural and functional connections with neurons and vascular elements. Astrocytic endfeet closely appose and encase blood vessels, and endothelial cells along with pericytes maintain well-organized morphology and normal physiological functions, supporting stable cerebral blood flow. **(B)** The Neurovascular Unit During Ischemic Stroke. Astrocytes, neurons, and microglia sustain damage or undergo cell death. Neuronal axons degenerate, microglia become polarized, and astrocytes become activated with enhanced proliferation. The BBB is disrupted, enabling the migration of circulating immune cells into the brain parenchyma, which results in inflammatory infiltration. Astrocytic endfeet are impaired and lose their tight association with blood vessels; endothelial cells and pericytes are damaged, leading to the breakdown of tight junctions and disorganized extracellular matrix deposition. Inflammatory cells accumulate and extravasate from blood vessels, while cerebral blood flow is significantly reduced or completely halted.

Therefore, understanding the mechanisms driving their phenotypic diversity, and exploring strategies to suppress harmful effects while promoting recovery, have become key research priorities. The following sections will discuss recent advances in the mechanisms and functions of astrocyte activation after IS, as well as potential approaches to modulate astrocytic responses for improved therapeutic outcomes (Figure 1).

## Intrinsic molecular mechanisms of astrocyte activation

### NF-κB signaling pathway

Following IS, astrocytes become activated and transition into reactive astrocytes, which significantly influence the progression and recovery of IS. Consequently, research into the mechanisms underlying astrocyte activation has been accelerating (O’Shea et al., 2024). Among these, the Toll-like receptor 4 (TLR4)–nuclear factor kappa-B (NF-κB) signaling pathway has been extensively studied. TLR4, a member of the Toll-like receptor family, is a transmembrane protein involved in immune responses that recognizes damage-associated molecular patterns (DAMPs) (Feldman et al., 2015; Sahoo, 2020). DAMPs released by cells in the infarcted area following IS are recognized by TLR4 receptors expressed on the astrocyte membrane. This leads to the recruitment of TRIF-related adaptor molecule (TRAM), which binds TRIF and subsequently activates TRAF3. TRAF3 then interacts with T3JAM to activate c-Jun N-terminal kinase (JNK), resulting in the phosphorylation of NF-κB and ultimately triggering astrocyte activation (Cheng et al., 2021). Meanwhile, after IS, the expression of triggering receptor expressed on myeloid cells-2 (TREM-2) on the cell membrane of astrocytes is increased. This upregulation inhibits the activation of astrocytes via the TLR4-NF-κB pathway and prevents their transition to the proinflammatory phenotype (Rosciszewski et al., 2018). Furthermore, both TLR4 and myeloid differentiation primary response protein 88 (MyD88) are upregulated after IS. It is suggested that TLR4 may also promote astrocyte activation through the MyD88-dependent pathway by inducing phosphorylation of the NF-κB subunit p65 (Jing et al., 2020). Liu et al. similarly demonstrated that enhanced JNK phosphorylation increases TLR4 expression and increases the phosphorylation level of p65 (Ser536), facilitating its nuclear translocation and regulating cellular activation (Liu et al., 2018). These findings indicate that TLR4 not only regulates astrocyte activation through multiple pathways but may also sustain and enhance the activation process via positive feedback.

Beyond the TLR4–NF-κB pathway, NF-κB can also be phosphorylated through other mechanisms to activate astrocytes. Following the onset of IS, the expression of low molecular mass peptide 2 (LMP2)—a subunit of the immunoproteasome—increases in both the cytoplasm and nucleus, whereas LMP2 knockout significantly inhibits NF-κB phosphorylation and attenuates astrocyte activation, while the specific mechanism by which the LMP2-NF-κB pathway regulates astrocyte activation remains to be further elucidated (Chen et al., 2015). Additionally, phosphatidylinositol 3-kinase-γ (PI3Kγ) is involved in astrocyte activation. Under hypoxic conditions, PI3Kγ degrades inhibitor of kappa B alpha (IKB-α), enhancing NF-κB phosphorylation and promoting astrocyte activation (Shang et al., 2019). The interaction between advanced glycosylation end products (AGEs) and their receptors is implicated in various diseases. After IS, astrocyte-derived S100B and increased plasma Aβ levels accumulate extracellularly, Which can both bind to the V domain of the receptor for advanced glycation end products (RAGE) on the cell membrane, leading to the recruitment and activation of DIAPH1, which ultimately results in NF-κB activation and astrocyte reactivity (Twarda-Clapa et al., 2022; Shen et al., 2021).

In summary, NF-κB plays a major role in the regulation of astrocyte activation, and multiple molecules can activate the NF-κB signaling pathway. However, alternative mechanisms also contribute to the modulation of astrocyte activation.

### STAT3 signaling pathway

The intracellular signaling pathway associated with Signal Transducer and Activator of Transcription 3 (STAT3) also plays a critical role in the activation of astrocytes. As a member of the STAT protein family, STAT3 can be phosphorylated and translocate into the nucleus to exert regulatory functions, similar to NF-κB (Gu et al., 2024). Following cerebral ischemia and hypoxia, increased phosphorylation of signal transducer and activator of transcription 3 (STAT3) in astrocytes promotes their inflammatory activation, while enhanced expression of αB-crystallin (CRYAB) and dopamine receptor D2 (DRD2) can inhibit astrocyte activation by suppressing STAT3 phosphorylation (Qiu et al., 2016). After the onset of IS, Lipocalin-2 (LCN2) is upregulated in astrocytes and shows co-localization with Glial fibrillary acidic protein (GFAP) (Zhang et al., 2018; Li et al., 2023). This upregulation enhances the activation of the JAK2/STAT3 signaling pathway and increases the expression of GFAP, indicating that the LCN2–JAK2–STAT3 signaling pathway is involved in regulating the proinflammatory activation of astrocytes (Zhang et al., 2018)

Given that astrocytes are in direct contact with neurons, endothelial cells, microglia, and other cell types (Alarcon-Martinez et al., 2023), their behavior in pathophysiological conditions can be influenced by secretions from other cells or even their own mediators. After cerebral ischemia, neuron-derived 17β-estradiol (E2) acts on astrocytes. Meanwhile, the expression of aromatase in astrocytes is upregulated, synergistically promoting E2 production and further inducing STAT3 phosphorylation as well as subsequent neuroprotective activation of astrocytes; furthermore, E2 inhibits fibroblast growth factor 2 (FGF2) secreted by neurons to sustain this activation process. Specifically, FGF2 can act on astrocytes and decrease the phosphorylation level of STAT3 through the FGF2–FGFR3–STAT3 signaling axis, thereby inhibiting the astrocyte activation process (Lu et al., 2020). In addition, Shang et al. demonstrated that after IL-6 binds to the soluble interleukin 6 receptor (sIL-6R) to form a complex, it activates STAT3 via phosphatidylinositol 3-kinase γ (PI3Kγ), thereby promoting the expression of GFAP and enhancing the inflammatory activation of astrocytes (Shang et al., 2019).

### MAPK signaling pathway

Mitogen-Activated Protein Kinase (MAPK) represents a family of kinase proteins, and its signaling pathway involves three major kinases—ERK (Extracellular Signal-Regulated Kinase), JNK, and p38—all of which are closely associated with cerebral ischemia (Patel et al., 2023; Cai et al., 2022; Xie et al., 2023). Natural aging itself can prime astrocytes to exhibit an activated state (Popov et al., 2023). Oxygen-glucose deprivation further enhances p38 MAPK activity, thereby promoting their activation (Revuelta et al., 2021). Tian et al. also demonstrated that activation of the MAPK signaling pathway upregulates GFAP expression (Tian et al., 2020). In addition to directly regulating astrocyte activation, the MAPK pathway can also modulate this process indirectly—for instance, by amplifying neuroinflammatory responses or through crosstalk with other signaling cascades. For example, Fas ligand (FasL) promotes JNK phosphorylation, which in turn stimulates inflammatory cell activation and the secretion of cytokines including TNF-α, IL-1β, and IL-6 (Tabarsa et al., 2022). Niu et al. found that genetic ablation of FasL reduces neutrophil infiltration and suppresses the activation of both microglia and astrocytes (Niu et al., 2012). Since these inflammatory cytokines are known to promote astrocyte activation (Shang et al., 2019), these findings suggest that Fas may indirectly modulate astrocyte reactivity by enhancing the production of pro-inflammatory mediators. Furthermore, the ASK1–JNK signaling axis, activated under ischemic and hypoxic conditions, can lead to NF-κB activation, thereby amplifying TLR4-mediated MAPK signaling and contributing to a sustained astrocyte response (Cheng et al., 2021).

## Non-coding RNAs regulate astrocyte activation

Non-coding RNAs (ncRNAs) also play a significant role in the regulation of astrocyte activation, with microRNAs (miRNAs), circular RNAs (circRNAs), and long non-coding RNAs (lncRNAs) being major focuses of research. miRNAs are a class of small non-coding RNAs that can specifically bind to target mRNAs, directly or indirectly regulating gene expression and influencing various physiological and pathological processes (Yates et al., 2013; Saliminejad et al., 2019). For instance, Dai, Kieran, and colleagues reported that elevated levels of miR-29c-5p and miR-210 in astrocytes following ischemia promote their activation (Dai et al., 2022; Kieran et al., 2022). In contrast, high expression of miR-488-3p inhibits astrocyte activation. However, after IS, increased levels of lncRNA NEAT1 bind to cytoplasmic miR-488-3p (via sequence complementarity: NEAT1 5′-CCTTCAA-3′ and miR-488-3p 3′-GGAAGUU-5′), thereby reducing miR-488-3p activity and promoting astrocyte activation (Zheng et al., 2023). Interestingly, ncRNAs also interact with multiple signaling pathways. Post-stroke reduction in miR-137 expression diminishes its inhibitory effect on Src gene expression—miR-137 binds to the 3′-UTR of Src mRNA (miR-137: 5′-UCGUUAU-3′; Src 3′-UTR: 3′-AGCAAUA-5′)—leading to increased cytoplasmic Src levels. As Src acts upstream of the MAPK pathway, its upregulation enhances the activity of SFKs kinases, which in turn phosphorylate p38, ERK1/2, and JNK, ultimately promoting astrocyte activation (Tian et al., 2020). Besides, mesenchymal stem cell-derived extracellular vesicles can deliver miR-125b-5p (5′-CAAUCCGAGUCCC-3′) into astrocytes, and this miRNA binds to TLR4 mRNA (3′-GUUAGGCUCAGGG-5′), thereby inhibiting TLR4 expression, reducing NF-κB activation and p65 phosphorylation, and ultimately suppressing astrocyte activation (Qiu et al., 2022).

Furthermore, both circRNAs and lncRNAs serve as molecular “sponges” or scaffolds, interacting with DNA, RNA, and proteins to modulate gene expression, protein function, and cellular differentiation (Zhang et al., 2023). Under ischemic conditions, Circular RNA HECTD1 (circHectd1) expression increases in astrocytes and competitively binds to MIR142 (circHectd1: 5′-CACUAC-3′; MIR142: 3′-GUGAUG-5′), reducing MIR142 availability. This indirectly elevates TIPARP levels, as TIPARP contains a conserved MIR142 binding site in its 3′-UTR that would otherwise suppress its expression. The increase in TIPARP promotes astrocyte activation (Han et al., 2018). Additionally, Zuo et al. demonstrated that circCDC14A, secreted by infiltrating neutrophils in the ischemic infarct area, also enhances astrocyte activation (Zuo et al., 2021). These findings illustrate that ncRNAs regulate astrocyte activation through multiple mechanisms and pathways, offering promising research directions for therapeutic intervention. However, the regulatory network controlling astrocyte activation is even more complex and warrants further in-depth investigation.

## Other ways to participate in activation regulation

Following the onset of IS, the accumulation of various intracellular and extracellular substances, enzyme activation, and ubiquitination processes significantly influence astrocyte activation (Rajput et al., 2020; Hong et al., 2023). For example, under ischemic and hypoxic conditions, decreased expression of Peroxisome Proliferator-Activated Receptor Alpha (PPARα) impairs autophagic flux, leading to the accumulation of medium and large autophagic vesicles and the ubiquitin-binding protein p62, which promotes astrocyte activation (Luo et al., 2023). Similarly, Wu et al. reported that cytoplasmic aggregation of Fused in Sarcoma (FUS) protein forms FUS inclusions wherein the prion-like domain plays a critical role in both aggregation and astrocyte activation, and these aggregates induce excessive and sustained autophagy to further mediate astrocyte reactivity (Wu et al., 2022).

Moreover, Ischemia-induced polarization of aquaporin-4 (AQP4) in astrocytic endfeet impairs the clearance efficiency of amyloid-β (Aβ) peptide in the brain parenchyma and glymphatic function (Li X. et al., 2024); on the one hand, the accumulated Aβ can activate astrocytes by binding to the RAGE (Shen et al., 2021), and on the other hand, AQP4-related glymphatic dysfunction leads to the accumulation of inflammatory factors, which may affect astrocyte activation (Shang et al., 2019). As noted earlier, increased TIPARP expression promotes astrocyte activation (Han et al., 2018) and also upregulates LC3B-II, enhancing autophagy and further reinforcing activation.

Enzymatic activation and physical stimuli also modulate astrocyte responses. Thrombin, generated via localized conversion of factor Xa, binds to protease-activated receptor 1 (PAR1) on astrocytes, thereby regulating gene expression and driving a shift toward the A2 reactive phenotype (Rajput et al., 2020). Additionally, pulsed blue laser light and activation of the Smad2/3 pathway via phosphorylation have been shown to upregulate GFAP expression, thereby modulating astrocyte activation (Suo et al., 2023; Luo et al., 2019).

**FIGURE 2:**
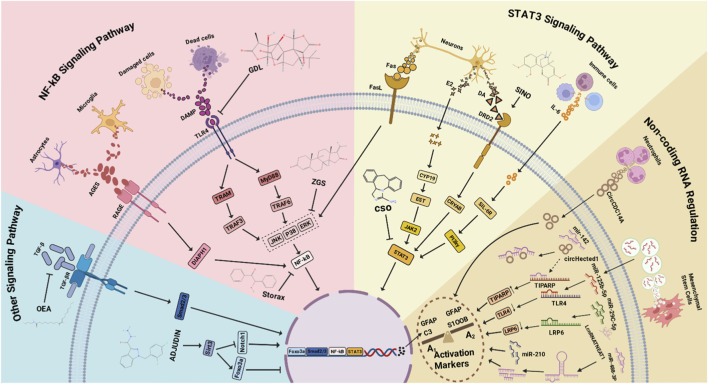
Multiple Mechanisms Regulating Astrocyte Activation. TGF-β binds to TGF-βR, inducing Smad phosphorylation and promoting astrocyte activation via the TGF-β/TGF-βR/Smad2/3 pathway. Upregulation of Sirt3 promotes Foxo3a expression and inhibits astrocyte activation. During IS, astrocytes and microglia secrete AGEs, which bind to RAGE, activating DIAPH1 and leading to NF-κB phosphorylation and nuclear translocation to regulate cell activation. During IS, DAMPs released from dead or damaged tissue cells bind to TLR4, activating TRAF3 via TRAM on one hand, and interacting with the adaptor protein MyD88 to activate TRAF6 on the other. Both pathways activate kinases (JNK, p38, ERK) and phosphorylate NF-κB to regulate cell activation. Neurons secrete various factors during IS that influence activation: Fas interacts with FasL, activating kinases (JNK, p38, ERK); E2 secretion leads to its conversion into EST via CYP19 in the cytoplasm, which subsequently activates JAK2; DA secretion binds to DRD2, activating CRYAB. All three pathways lead to STAT3 phosphorylation and nuclear translocation, regulating cell activation. Various infiltrating immune cells during IS secrete IL-6, which complexes with cytoplasmic sIL-6R and phosphorylates STAT3 via PI-3Kγ, leading to STAT3 nuclear translocation and cell activation. Neutrophils secrete circCDC14A, which enters astrocytes and influences their activation. During IS, increased circHectd1 competes with TIPARP to bind miR-142, reducing miR-142 levels and indirectly increasing TIPARP levels, thereby promoting activation. Mesenchymal stem cells (MSCs) deliver miR-125b-5p into astrocytes via extracellular vesicles (EVs). miR-125b-5p binds to TLR4 mRNA, reducing its levels and modulating the TLR4 signaling pathway, thereby affecting cell activation. Increased miR-29c-5p binds to LRP6 mRNA, reducing its levels and affecting cell activation. miR-210 levels increase after IS, promoting astrocyte activation. After IS, lncRNA NEAT1 levels increase, binding to cytoplasmic miR-488-3p and reducing its expression, thereby promoting astrocyte activation.

In summary, we have outlined multiple pathways involved in the regulation of astrocyte activation under ischemic conditions and highlighted several associated alterations whose underlying mechanisms remain incompletely understood. It is clear, however, that following IS, astrocytes undergo activation in response to diverse stimuli, resulting in morphological and functional changes that enable these cells to exert complex and often dual roles in stroke pathology and recovery (Figure 2).

## Role of astrocytes after activation

After the onset of IS, astrocytes are activated by a variety of stimuli and can secrete cytokines to participate in the inflammatory response through pathways such as NF-κB, STAT3, and MAPK (Rosciszewski et al., 2018; Qiu et al., 2016; Tian et al., 2020). On one hand, reactive astrocytes can secrete inflammatory factors and chemokines (e.g., IL-1β, TNF-α, IL-6, and CXCL10) to exacerbate the inflammatory response, induce cell death, and thereby slow down disease recovery (Li X. et al., 2024; Park et al., 2020; Yang et al., 2022; Wang et al., 2024). On the other hand, reactive astrocytes can inhibit inflammation by increasing the expression of IL-1ra, IL-10, and Arg1, and exert a protective effect to reduce brain damage by promoting the secretion of BDNF, IGF-1, and GLT-1 (Lu et al., 2020; Kieran et al., 2022; Wang and Li, 2023). In addition, with respect to the BBB, reactive astrocytes may disrupt the BBB by decreasing the expression of tight junction proteins (claudin-5, ZO-1) and increasing the expression of P-gp or MMP9 (Kim et al., 2020; Jing et al., 2020; Suo et al., 2023; Wang et al., 2020). However, reactive astrocytes can also maintain BBB integrity and promote angiogenesis by inhibiting MMP2 and MMP9 via IL-10 or increasing the expression of VEGF and FGF2 (Suo et al., 2023; Li et al., 2022; Feng et al., 2023; Marushima et al., 2020). Therefore, intervening in the phenotypic transition of astrocytes represents a viable research direction for the treatment of ischemic stroke.

## Astrocyte activation as a therapeutic target in ischemic stroke: Mechanisms and opportunities

### Multimodal rehabilitation in ischemic stroke: From mechanisms to functional recovery

As discussed above, activated astrocytes can exert diverse effects, and research has consistently focused on mitigating their detrimental roles while enhancing their beneficial functions. Several strategies have shown promise in achieving this goal, including rehabilitative therapies, naturally derived activating components, and pharmacological interventions.

Repetitive Transcranial Magnetic Stimulation (rTMS) has been applied in clinical treatment following IS. rTMS helps preserve the integrity of existing vasculature to prevent disease progression and reduces BBB hyperpermeability. Concurrently, a reduction in the volume of peri-infarct reactive astrocytes has been observed, along with increased expression of PDGFRβ and VEGF in astrocytes, creating a favorable environment for tissue repair and angiogenesis (Zong et al., 2020). Furthermore, rTMS modulates astrocyte function by downregulating the expression of C3, iNOS, and IL-12, while promoting the expression of S100A10 and Arg1, thereby exerting neuroprotective effects. Co-culture studies further indicate that rTMS indirectly reduces neuronal apoptosis through astrocyte stimulation, promotes neuronal synaptogenesis, increases axonal density and synaptic plasticity, and ultimately supports cognitive recovery (Hong et al., 2020). Additionally, rTMS promotes a protective phenotypic shift in astrocytes, enhances astrocyte-vascular coupling, and helps maintain vascular density and continuity (Zhang et al., 2025).

Acupuncture, particularly in its modern form such as Electroacupuncture (EA), has long held a significant place in medical practice. EA exerts neuroprotective effects by activating cannabinoid receptor 1 (CB1R) on astrocytes, thereby modulating extracellular glutamate levels (Yang et al., 2021; Liu et al., 2023). It also mitigates astrocyte activation and inhibits ROS production, reducing BBB disruption and alleviating cerebral edema (Jung et al., 2016). Moreover, EA influences energy metabolism in IS by significantly increasing astrocyte numbers and upregulating the expression of monocarboxylate transporter 1 (MCT1), which enhances lactate shuttle activity between astrocytes and neurons, supporting neuronal survival (Lu et al., 2015).

Other rehabilitative approaches have also been reported in IS treatment. Hypothermia therapy reduces astrocyte cell death and enhances cell viability (Lyden et al., 2019), while also downregulating MMP-2 and MMP-9 expression in astrocytes to protect BBB integrity (Lee et al., 2005). Normobaric hyperoxia (NBO) therapy has been shown to facilitate stroke recovery by attenuating astrocyte activation and reducing glial scar formation, accompanied by significant improvement in motor function (Esposito et al., 2013). NBO also upregulates Connexin 43 (Cx43) and GPX4 expression in astrocytes, reducing cytochrome c-mediated apoptosis and restoring intercellular connectivity, thereby decreasing neuronal injury (Qi et al., 2022). Regular long-term physical exercise increases the expression of STAT3 and Gpc6 after IS onset, promotes the transition of astrocytes to a neuroprotective phenotype, reduces infarct volume, and enhances functional recovery. It also increases the synaptic density between astrocytes and neurons, providing support for neural rehabilitation and motor rehabilitation (Chen et al., 2021). Combination therapies, such as EA coupled with exercise, facilitate astrocyte phenotypic modification, suppress the secretion of pro-inflammatory cytokines (TNF-α and IL-1β), reduce ROS levels, and decrease cell death (Li Z. et al., 2020).

The core advantage of rehabilitative therapies resides in their capacity to simultaneously target multiple components of the neurovascular unit, thereby producing synergistic effects. Unlike invasive procedures such as craniotomy, interventions like rTMS, EA, and exercise therapy are non-invasive or minimally invasive, associated with reduced trauma and lower risk of infection (Galvin et al., 2015). Moreover, these approaches avoid the systemic burden associated with pharmacotherapies—such as hepatorenal toxicity, drug interactions, and potential adverse effects—thereby facilitating clinical translation (Tan et al., 2025). However, further optimization is still required, including standardization of stimulation parameters and treatment locations for rTMS and EA. Additionally, treatment efficacy may be influenced by patient age, stroke severity, and genetic background, underscoring the need for personalized therapeutic strategies (Hegedus et al., 2022).

### Natural plant-derived bioactive compounds: Therapeutics for ischemic stroke

Naturally derived compounds extracted from medicinal plants can exert specific physiological or therapeutic effects in the human body. Many of these bioactive components are also capable of modulating astrocyte function, thereby promoting recovery after IS.

Tetramethylpyrazine (TMP), an active ingredient from *Ligusticum chuanxiong*, reduces astrocyte damage and cell death via aquaporin 4 (AQP4) and connexin 43 (Cx43), and promotes the secretion of FGF2 by astrocytes to support neurogenesis and angiogenesis, thereby contributing to the reconstruction of the cerebral microenvironment (Feng et al., 2023). Buyang Huanwu Decoction, containing multiple active compounds such as ferulic acid, astragaloside IV, and paeoniflorin, stimulates astrocytes to release BDNF, thereby protecting neurovascular unit integrity and facilitating neurovascular remodeling, while also upregulating MCT4 expression to enhance lactate shuttle activity, supplying energy to neurons and reducing neuronal damage, and concurrently inhibiting glial scar formation to create a favorable environment for recovery (Li M.-C. et al., 2024). Z-Guggulsterone (Z-GS), derived from myrrh, enhances astrocyte viability and reduces apoptosis. It suppresses the TLR4/NF-κB pathway to inhibit pro-inflammatory cytokine release, while promoting the expression of anti-inflammatory mediators such as IL-10 and TGF-β1. Notably, even at a high dose of 1,600 mg/kg, no adverse effects were observed, indicating a favorable safety profile (Liu et al., 2018). Celastrol, a natural bioactive compound, binds to Nedd4 in astrocytes, disrupting the Nedd4–Nrf2 interaction and increasing Nrf2 protein levels. This attenuates oxidative stress, prevents astrocyte apoptosis and glial scar formation, and indirectly reduces neuronal injury (Hong et al., 2023). Ginsenoside Rb1 inhibits astrocyte activation and apoptosis by reducing ROS production. It also facilitates the transfer of functional mitochondria to neurons, mitigating neuronal damage and promoting neurological recovery. Additionally, Rb1 decreases glutamate-induced excitotoxicity in astrocytes and promotes the secretion of neurotrophic factors (NGF, BDNF, and GDNF), collectively supporting neuronal survival (Ni et al., 2022).

An extract from *Alpinia oxyphylla* Miq [Yi Zhi Ren (YZR)] significantly suppresses JNK activation and inhibits the ASK1/JNK/NF-κB pathway, leading to reduced astrocyte activation and inflammatory cytokine expression. This downregulation of inflammatory signaling helps limit cerebral infarction and preserve neurological function (Cheng et al., 2021). Storax exerts protective effects by inhibiting NF-κB activation, thereby reducing astrocyte reactivity, neuroinflammation, and cytotoxicity (Zhou et al., 2021). The aqueous extract of *Ruta graveolens* effectively attenuates inflammatory activation of astrocytes in the cortex and striatum, increases BDNF expression, reduces brain injury, improves neurological function, and suppresses inflammation (Campanile et al., 2022). Ginkgo biloba extract (EGB) inhibits LCN2 overexpression and the JAK2/STAT3 pathway in astrocytes, thereby suppressing astrocyte activation and the release of inflammatory factors, which in turn reduces infarct size and neuronal damage (Zhang et al., 2018). Ginkgolide (GDL) treatment inhibits astrocyte activation and the expression of IL-1β and TNF-α, enhances cell viability, and reduces peri-scar astrocyte reactivity, creating favorable conditions for post-stroke recovery (Li X. et al., 2020). Cottonseed oil (CSO) inhibits TLR4-mediated microglial activation and TNF-α secretion, thereby suppressing NF-κB-driven astrocyte activation. It reduces the release of inflammatory cytokines and promotes a protective astrocyte phenotype, ultimately alleviating IS-induced neuronal injury (Liu et al., 2020).

In summary, most natural bioactive compounds modulate astrocytes through multiple targets, inhibit inflammatory pathways, attenuate neuroinflammation, and provide neurovascular protection. They also enhance lactate-mediated energy supply, reduce glial scar formation, and remodel the neural microenvironment. These compounds demonstrate good safety profiles and wide therapeutic windows. Rather than simply inhibiting astrocyte activity, they induce functional modulation, showing promise as next-generation neuroprotective agents (Zeng et al., 2023). However, most current evidence is derived from preclinical studies. Challenges remain in elucidating their *in vivo* metabolism, BBB permeability, and active components in compound formulations (Li et al., 2025). Future research should focus on conducting large-scale, multicenter randomized controlled trials to validate their efficacy, as well as developing targeted delivery systems such as nanocarriers to improve their bioavailability and specificity.

### Agonists and small-molecule therapeutics in ischemic stroke

In recent years, a variety of pharmacological interventions targeting specific molecules—including both natural and synthetic small-molecule agonists as well as biologics—have been demonstrated to effectively modulate astrocyte reactivity, thereby conferring neuroprotection, anti-inflammatory effects, and functional recovery after IS.

The dopamine D2 receptor (DRD2) agonist Sinomenine (Sino) inhibits astrocyte activation in a dose-dependent manner, directly reducing the expression of multiple inflammatory cytokines and attenuating neuroinflammatory damage (Qiu et al., 2016). Oleoylethanolamide (OEA), an endogenous agonist of PPARα, promotes PPARα nuclear translocation and exerts multiple beneficial effects: it suppresses astrocyte activation and migration, mitigating glial scar formation, while concurrently upregulating neuroplasticity-related proteins to promote neural regeneration and functional recovery (Luo et al., 2019). Adjudin (a potential Sirt3 activator) exerts its effects through a dual mechanism: it enhances the activity and expression level of Sirt3. On the one hand, Sirt3 upregulates the expression of Foxo3a, thereby augmenting the inhibitory effect of the Sirt3/Foxo3a signaling pathway on astrocyte activation; on the other hand, it inhibits the transcriptional activity of Notch1, thus attenuating the promoting effect of the Sirt3/Notch1 signaling pathway on astrocyte activation. This synergistic effect reduces astrocyte proliferation and alleviates glial scar formation, and these effects collectively support functional recovery. (Yang et al., 2017). Afobazole, an agonist of σ1/σ2 receptors, reduces post-stroke astrocyte activation and reactive proliferation, preventing glial scar formation. It also attenuates nitrosative stress, providing additional neuroprotective benefits (Katnik et al., 2016).

Exogenous administration of interleukin-33 (IL-33) polarizes helper T cells and macrophages toward an anti-inflammatory M2 phenotype, thereby increasing the secretion of anti-inflammatory factors such as IL-4, which indirectly suppresses astrocyte activation and cytokine release (Korhonen et al., 2015). Intranasal delivery of complement C3a enables rapid entry into the brain, where it binds to the C3a receptor (C3aR) on astrocytes, directly inhibiting their activation and proliferation. This approach reduces glial scarring and neuroinflammation, while upregulating neurotrophic factors such as IGF1 and Thbs4 to promote neural plasticity and synaptic recovery (Stokowska et al., 2023; Jj and Rg, 2012).

Current research reveals a new therapeutic paradigm: precise modulation of astrocyte activity and the immune microenvironment can synergistically alleviate neuroinflammation, weaken the glial scar barrier, and provide neurotrophic support to facilitate neural repair. Although the drug targets are diverse (Sino, C3a, IL-33, Adjudin), they collectively demonstrate the ability to regulate astrocyte activation, highlighting the potential of combinatory strategies to improve the cerebral microenvironment (Wardlaw et al., 2023). Many of these agents are derived from natural or endogenous sources, offering favorable safety profiles. The intranasal administration of C3a allows efficient BBB penetration, enhancing clinical feasibility. Compounds such as afobazole remain effective even when administered beyond the acute phase, showing particular promise for patients who miss the conventional treatment window (Sang et al., 2021). However, most evidence remains preclinical (Table 1). Optimal dosing timing, combinatory mechanisms, and targeted delivery technologies require further refinement. Future efforts should focus on improving drug bioavailability, developing precision delivery systems, and systematically evaluating the efficacy and safety of combination therapies in well-designed clinical trials.

**TABLE 1:** Basic data and efficacy of treatment.

Interventions	Resea	Disease model	Treatment parameters	Time of treatment initiation	Course	Site/mode of interver	Effects and phenotypes	Location
repetitive transcranial magirat		photothrombotic	3 pulses of 50 Hz repeated et	3 h postoperatively	6 days once a day	infarcted hemisphere	Protects the blood-brain barrier, protects the vasculature, promotes angiogenesis, protects	Zong et al. (2020)
repetitive transcranial magirat		middle cerebral artery occlusion	10Hz, 10min	1 day after surgery	1w, once a day	infarcted hemisphere	Reduces cell death, promotes synapse formation, improves cognitive function	Hong et al. (2020)
repetitive transcranial	magimice	middle cerebral artery occlusion	10 bursts at 50 Hz repeated 2	2 days after surgery	1 or 2w, twice	infarcted hemisphere	Improves nerve motor function, protects blood vessels, reduces cell death, increases cen	Zhang et al. (2025)
electroacupuncture	mice	middle cerebral artery occlusion	1 mA and frequency of 2/15 F	7 days postoperatively	3 days once a day	Bailnu acupomt	Reduce infarct volume reduce nerve damage	Yang et al. (2021)
electroacupuncture	rat	middle cerebral artery occlusion	15Hz, 20min	1 day after surgery	3 days once a day	Tongtian and Luoque	Reduce nerve damage, reduce cell death, reduce infarct area	Liu et al. (2023)
electroacupuncture	mice	middle cerebral artery occlusion	lmA, 2Hz, 20min	3 days before surgery	Once day	Bailnu and Dazlnu	Protects the blood-brain barrier, reduces oxidative stress, prevents cerebral edema	Jung et al. (2016)
electroacupuncture	rat	middle cerebral artery occlusion	1 mA, 2/15 Hz, 20 mm	2 h postoperatively	1w, once a day	Neiguan and Quchi	Restores nerve damage, increases the concentration of lactic acid	Lu et al. (2015)
Therapeutic hypothermia	rat	middle cerebral artery occlusion	32.5 °C, 2 h or 4 h	Oh postoperatively	once	whole body	Reduce infarct area. reduce nerve damage, reduce cell death	Lyden et al. (2019)
Therapeutic hypothermia	rat	middle cerebral artery occlusion	33 °C, 2 h or 4 h or 24 h	Oh postoperatively	once	whole body	Protects the blood-brain barrier	Lee et al. (2005)
Normobaric oxygen	rat	middle cerebral artery occlusion	100%O2, 85min	5min postoperatively	once	whole body	Reduce the infarct area improve motor function	Esposito et al. (2013)
Normobaric oxygen	rat	middle cerebral artery occlusion	100%O2, 90min	10min after surgery	once	whole body	Reduce infarct area, inhibit oxidative stress, reduce cell death	Qi et al. (2022)
running	mice	middle cerebral artery occlusion	10 m/min 60 mm	—	2w, once a day	whole body	Promotes synapse formation, cell proliferation, reduces glial scarring	Chen et al. (2021)
scalp acupuncture and exerrat		middle cerebral artery occlusion	1-3 days,5 m/min; 4–14 days,10 m/mm	1 day after surgery	3w, once a day	whole body/Bailnu pc	Reduce the infarct area, reduce nerve damage, reduce inflammatory response	Li et al. (2020a)
2,3,5,6-Tetramethylpyrazine	rat	middle cerebral artery occlusion	10 or 20 or 40 mg/kg	4 h postoperatively	2w, once a day	Intraperitoneal mjecti	Reduce the infarct area, improve cerebral perfusion, protect neurovascular units	Feng et al. (2023)
Buyang Huanwu Decoction		middle cerebral artery occlusion	16.6 g/kg	24 h postoperatively	30days, once a da	Gavage	Reduce the infarct area, reduce brain tissue damage, promote angiogenesis, promote syr	Li et al. (2024b)
Z-Guggulsterone	rat	middle cerebral artery occlusion	30 or 60 mg/kg	Oh postoperatively	3days, once a day	take orally	Inhibit inflammatory response, increase cell viability, reduce cell death, reduce infarct vo	Liu et al. (2018)
Celastrol	mice	transient middle cerebral artery occhr	4.5 mg/kg	Oh postoperatively	once	Intraperitoneal injecti	Reduces cerebral infarction, inhibits oxidative stress, improves neurological motor functi	Hong et al. (2023)
Ginsenoside Rbl	mice	Photochemical cerebral thrombosis	100 mg/kg	Id before surgery and 2 days a	3 times	Intraperitoneal mjecti	Reduces cell death, promotes neuronal recovery, reduces oxidative stress	Ni et al. (2022)
Alpinia oxyphylla Miq	rat	middle cerebral artery occlusion	0.2 or 0.4 or 0.8 g/kg	Oh postoperatively	once	Intraperitoneal injecti	Reduce infarct area improve neurological motor function	Cheng et al. (2021)
Storax (SuHeXiang)	rat	middle cerebral artery occlusion	0.4 g/kg	1h, 3h, and 6 h postoperative	3 times	Gavage	Reduce neurological deficits, reduce infarct area, increase cerebral vascular synaptic de	Zhou et al. (2021)
Ruta graveolens water extra		middle cerebral artery occlusion	10 or 30 or 10 mg/kg	100min and 300min after SL	twice	Intraperitoneal injecti	Reduce neurological deficits reduce the infarct area	Campanile et al. (2022)
Ginkgo biloba extract	rat	Microsphere-induced cerebral embolis	7.5 or 15 mg/kg	Postoperative 1day and 3 d	2 times	Duodenal injection	Reduce infarct area reduce nerve damage	Zhang et al. (2018)
Ginkgo biloba L	rat	middle cerebral artery occlusion	1.25 mg/kg	1 h postoperatively	3days, twice a day	intravenous injection	Reduce infarct area, reduce cerebral edema, improve nerve damage, inhibit inflammator	Li et al. (2020b)
Cottonseed oil	rat	middle cerebral artery occlusion	1.3 mL/kg	—	3w, once a day	subcutaneous injectio	Reduce the infarct area, reduce neuronal damage, relieve cerebral edema	Liu et al. (2020)
sinomenine	mice	middle cerebral artery occlusion	10 or 20 mg/kg	6 h,1d, and 2 days after surgery	3 times	Intraperitoneal injecti	Reduces infarct volume, reduces cell death, mitigates nerve damage, inhibits inflammato	Qiu et al. (2016)
Oleoylethanolamide	mice	middle cerebral artery occlusion	10 mg/kg	Oh postoperatively	12days, once a da	take orally	Reduces cell migration, reduces glial scarring, restores motor function	Suo et al. (2023)
adjudin	mice	transient middle cerebral artery occhr	50 mg/kg	lw and 5w postoperatively	2w, twice a da	Intraperitoneal injecti	Reduces cell proliferation, reduces glial scarring, promotes angiogenesis	Yang et al. (2017)
afobazole	rat	middle cerebral artery occlusion	3 mg/kg	1 day after surgery	3 days 3 times a d	Intraperitoneal injecti	Enhance cell viability, reduce cell death, inhibit inflammatory responses	Katnik et al. (2016)
recombinant IL-33	mice	middle cerebral artery occlusion	1 µg/mouse	Oh postoperatively/lw posto	Once/twice	Intraperitoneal injecti	Reduce the infarct area improve motor function	Korhonen et al. (2015)
C3a	mice	cortical photothrombosis	1.13ug/kg	1 day after surgery	2w/3w, once a	Nasal administration	Improve motor function, reduce infarct area, inhibit inflammatory response, promote syn	Stokowska et al. (2023)

### Synergistic combination therapy: Enhancing targeted delivery and efficacy of therapeutics via rehabilitation-based priming

As previously discussed, although physical rehabilitation therapies (such as EA and rTMS), natural active compounds, and targeted pharmacological agents each possess distinct advantages, they also face limitations including inefficient delivery, poor targeting, and low bioavailability (Romera et al., 2025). Combining these interventions offers a novel multi-modal and synergistic therapeutic strategy: using non-invasive or minimally invasive rehabilitation techniques as priming modalities to modulate the intracerebral microenvironment can enhance the efficiency and specificity of subsequently administered bioactive compounds or drugs.

EA, with its inherent electrical stimulation properties, can be leveraged to promote drug penetration across the BBB via electroporation-based techniques, thereby improving drug delivery to lesioned areas (Yin et al., 2025). rTMS is known to transiently and reversibly increase BBB permeability under specific magnetic stimulation parameters (Zong et al., 2020). This effect may stem from the modulation of tight junctions in vascular endothelial cells or through influencing the expression and distribution of aquaporin-4 (AQP4) on astrocytic end-feet (Jung et al., 2016). Within a specific time window after rTMS or EA application, BBB permeability is temporarily and moderately enhanced. Administering natural active ingredients (tetramethylpyrazine, TMP or ginsenoside Rb1) or therapeutic drugs (complement C3a or afobazole) during this period allows these agents to cross the BBB more efficiently, achieve higher effective concentrations at the target site, and fully exert their neuroprotective, anti-inflammatory, and pro-repair effects. Rehabilitation therapies essentially open a temporal “window of opportunity” for drugs, which in turn utilize this window for enhanced brain delivery. This approach not only improves therapeutic efficacy but may also reduce potential systemic side effects by lowering the required drug dosage.

Advances in nanocarrier research demonstrate that encapsulating natural compounds or drugs within smart nanoparticles can confer additional targeting and controlled-release capabilities. For example, nanoparticles can be engineered with magnetothermal responsiveness (Feng et al., 2016) and surface-functionalized with targeting moieties—such as Celastrol for targeting Nedd4 or antibodies against astrocyte-specific surface antigens like ACSA-2—enabling initial accumulation in the lesion area (Hong et al., 2023; Bae and Park, 2020; Son et al., 2025). When exposed to rTMS-induced magnetic fields, these nanoparticles generate local heat, leading to phase change or structural disruption and subsequent drug release (Song et al., 2020). Here, rTMS not only provides therapeutic benefits but also acts as an external trigger for targeted drug release. Similarly, techniques such as focused ultrasound combined with microbubbles (FUS-MB) have been shown to open the BBB and simultaneously activate drug release from ultrasound-sensitive carriers (Cornelssen et al., 2024). The combination of rTMS and magnetothermal nanocarriers represents a promising pathway for achieving spatially and temporally controlled drug release in the brain.

Beyond modulating the BBB, rehabilitation therapies can remodel the local cerebral microenvironment. Inflammatory factors, reactive oxygen species, and cellular damage may degrade drug integrity and impair target cell responsiveness, reducing treatment efficacy (Gu et al., 2022). Pathological conditions can lead to diminished or absent expression of target proteins, while inflammatory mediators may destabilize therapeutic compounds (Wan et al., 2025). By attenuating neuroinflammation, improving metabolic function, and restoring vascular integrity, rehabilitation interventions can create a microenvironment that favors drug stability and target engagement. Both rTMS and EA have been shown to reduce neuroinflammation, improve energy metabolism, and promote vascular repair and remodeling (Zong et al., 2020; Lu et al., 2015). These changes collectively shift the cerebral milieu toward a protective and reparative state, significantly improving the pharmacokinetics and pharmacodynamics of subsequently administered drugs. The application of smart hydrogels that respond to ROS levels—releasing anti-inflammatory drugs such as minocycline during peak inflammation—demonstrates how the microenvironment itself can be harnessed to trigger on-demand drug release, thereby enhancing therapeutic precision and efficiency (Zhou et al., 2025).

The core concept of this combined strategy is to transform rehabilitation therapy from a standalone treatment into a foundational approach that optimizes the environment for drug action and serves as a trigger for precise drug release. This multi-modal, temporally coordinated therapeutic paradigm has the potential to overcome current limitations in drug delivery and efficacy, offering an innovative and highly translatable solution for the treatment of IS.

## Conclusions and prospects

Following IS, astrocytes are activated into a reactive phenotype, playing a complex and dual role in disease progression. On one hand, they exacerbate brain injury through multiple mechanisms, including modulating excessive cellular activation, promoting the release of inflammatory factors, amplifying neuroinflammation, forming glial scars, and inducing cell death. On the other hand, they exert protective effects by secreting neurotrophic factors, facilitating angiogenesis, maintaining BBB integrity, and supporting neuronal metabolism. Therapeutic strategies targeting this activation process show considerable promise. Interventions such as rehabilitative therapies, natural plant-derived compounds, and pharmacological agents can modulate astrocyte phenotypes, suppress detrimental responses, and enhance protective functions, thereby offering a multi-target therapeutic approach to promote neural repair.
